# The Q336H MAPT Mutation Linked to Pick’s Disease Leads to Increased Binding of Tau to the Microtubule Network *via* Altered Conformational and Phosphorylation Effects

**DOI:** 10.3389/fnmol.2020.569395

**Published:** 2020-12-02

**Authors:** Giacomo Siano, Mariachiara Micaelli, Arianna Scarlatti, Valentina Quercioli, Cristina Di Primio, Antonino Cattaneo

**Affiliations:** ^1^Laboratorio di Biologia BIO@SNS, Scuola Normale Superiore, Pisa, Italy; ^2^Istituto di Neuroscienze, CNR, Pisa, Italy

**Keywords:** *Tau mutation*, aggregation, Pick’s disease, FRET, FRAP

## Abstract

Tauopathies are neurodegenerative disorders characterized by Tau aggregation. Genetic studies on familial cases allowed for the discovery of mutations in the MAPT gene that increase Tau propensity to detach from microtubules and to form insoluble cytoplasmic Tau aggregates. Recently, the rare mutation Q336H has been identified to be associated with Pick’s disease (PiD) and biochemical analyses demonstrated its ability to increase the microtubules (MTs) polymerization, thus revealing an opposite character compared to other Tau mutations studied so far. Here we investigated the biophysical and molecular properties of Tau^Q336H^ in living cells by the employment of the conformational Tau biosensor CST. We found that this mutation alters Tau conformation on microtubules, stabilizes its binding to tubulin, and is associated with a paradoxical lower level of Tau phosphorylation. Moreover, we found that this mutation impacts the cytoskeletal complexity by increasing the tubulin filament length and the number of branches. However, despite these apparently non-pathological traits, we observed the formation of intracellular inclusions confirming that Q336H leads to aggregation. Our results suggest that the Tau aggregation process might be triggered by molecular mechanisms other than Tau destabilization or post-translational modifications which are likely to be detrimental to neuronal function *in vivo*.

## Introduction

Tauopathies are a group of heterogeneous pathologies characterized by the dysfunction and aggregation of Tau protein. While the pathological mechanisms involved in the sporadic disease are difficult to be studied, familial tauopathies are associated with genetic alterations that can be more easily addressed for clinical investigation (Alonso et al., 2008; Wolfe, 2009; Arendt et al., 2016).

Mutations in the MAPT gene, encoding Tau protein, are usually associated with familial disease indeed, up to now, almost 50 mutations have been identified in patients affected by different tauopathies (Strang et al., 2019).

Most of these mutations are localized in the microtubule-binding domain (MTBD) or in proximal regions and determine alterations in the splicing of Tau mRNA thus leading to the unbalance of 3R/4R Tau isoforms. In several cases, MAPT mutations reduce Tau binding to microtubules (MTs) and the tubulin polymerization, a pathological effect with dramatic consequences on the cytoskeletal functions. Moreover, some mutations increase Tau sensitivity to post-translational modifications, in particular the hyperphosphorylation followed by the formation of toxic amyloidogenic aggregates. All these alterations ultimately lead to synaptic dysfunction and neuronal cell death (Hasegawa et al., 1998; Dayanandan et al., 1999; Goedert and Jakes, 2005; Fischer et al., 2007; Alonso et al., 2008; Wolfe, 2009; Iqbal et al., 2010; Strang et al., 2019). Tau mutations are a valuable tool to reproduce and study *in vitro* and *in vivo* the pathological mechanisms associated with Tau destabilization and aggregation.

Recently, two novel missense mutations in exon 12, Q336R and Q336H, have been identified in individuals with a familial tauopathy, the Pick’s disease (PiD), characterized by frontotemporal atrophy, neurodegeneration, gliosis, and Tau aggregates (Munoz et al., 2003; Yamakawa et al., 2006; Arendt et al., 2016). Previous *in vitro* studies reported that Tau proteins bearing these mutations can undergo pathological aggregation but, contrary to all other mutants they increase the tubulin polymerization and show a higher affinity for MTs (Pickering-Brown et al., 2004; Tacik et al., 2015), suggesting alternative Tau-dependent mechanisms for the onset of Tau pathology.

Thus, these mutants stand out from all other Tau mutants as having distinct properties and could teach us a lot about the mechanisms of Tau mutations in causing neurodegeneration. For this reason, here we investigate the peculiar biophysical and molecular properties of Tau^Q336H^ mutant by employing the CST, a conformational full-length Tau biosensor, allowing us to study the Tau behavior in physiological and pathological conditions in live cells (Di Primio et al., 2017; Siano et al., 2019a).

## Materials and Methods

### Mutagenesis

The Conformational Sensitive Tau (CST^WT^) plasmid, already available in the lab (Di Primio et al., 2017), has been mutagenized by the Site-Directed Mutagenesis Kit Q5 (New England BioLabs) according to the manufacturer’s instructions to obtain the CST^Q336H^. The primers employed are: Fwd 5′-AGGAGGTGGCCACGTGGAAGT-3′; Rev 5′-GGTTTATGATGGATG TTGCC-3′.

### Cell Culture

HeLa cells were maintained in Dulbecco’s modified Eagle’s medium (DMEM; GIBCO) supplemented with 10% FBS. The day before the experiment cells were seeded at 10^5^ cells in six-well plates or in Willco dishes (Willcowells). Lipofection was carried out with Effectene (QIAGEN) according to the manufacturer’s instructions.

### Western Blot

For western blot experiments, total protein extracts were prepared in lysis buffer supplemented with protease and phosphatase inhibitors. Proteins were quantified by BCA (Thermo Fisher Scientific). Twenty microgram of total protein was loaded for each sample. Proteins were separated by SDS–PAGE and electro-blotted onto Hybond-C-Extra (Amersham Biosciences) nitrocellulose membranes. Membranes were blocked with 5% skimmed milk powder in TBS, 0.1% Tween 20. Primary antibodies for WB: mouse anti-Tau (Tau13) 1:1,000 (Santa Cruz Biotechnology); rabbit anti-pTau (pAT8) 1:500 (Thermo Fisher Scientific); rabbit anti-pTau (Ser262) 1:500 (Thermo Fisher Scientific); mouse anti-pTau (Thr231) 1:500 (Thermo Fisher Scientific); rabbit anti-tubulin 1:5,000 (Abcam). Secondary antibodies for Western blot analysis were HRP-conjugated anti-mouse and, anti-rabbit 1:1,000 (Santa Cruz Biotechnology). Western blot quantification has been performed using ImageJ software.

### Immunofluorescence

For immunofluorescence (IF) experiments, cells were fixed with ice-cold 100% methanol for 5 min. Cell membranes were permeabilized (0.1% Triton-X100 in PBS) and samples were blocked (1% BSA in PBS) and incubated with the primary antibody (O/N, 4°C) and with fluorophore-conjugated secondary antibodies (1 h, RT). Slides were mounted with VECTASHIELD mounting medium (Vector Laboratories). Primary antibodies for IF: rabbit anti-tubulin 1:500 (Abcam). Secondary antibody: goat anti-rabbit Alexa Fluor 633 1:500 (Life Technologies). For K114 staining, cells were fixed and permeabilized as described above. Samples were incubated with 1 μM K114 (Sigma–Aldrich) for 10 min and slides were mounted with VECTASHIELD.

### Tau Seeding and Treatment

Recombinant heparin-assembled Tau fibrils were prepared as previously described (Siano et al., 2019a). For this assay, we employed P301S seeds that are a well-known and reliable reagent to prime Tau aggregation. Cells were plated in glass-bottom dishes and the following day the CST^WT^ and the CST^Q336H^ were transfected. 1.2 μg of Tau fibrils were delivered to cells with 2 μl of Lipofectamine 2000 transfection reagent diluted in 300 μl of Opti-MEM Reduced Serum Medium (Gibco). Cells were treated for 2 h, then DMEM low glucose was added back to HeLa. After 72 h imaging experiments have been performed.

### Image Acquisition, FRET and FRAP

Images were acquired with the TCS SP2 laser-scanning confocal microscope (Leica Microsystems) equipped with a galvanometric stage using a 63/1.4 NA HCX PL APO oil immersion objective. A heated and humidified chamber was used for live imaging experiments to maintain a controlled temperature (37°C) and CO_2_ (5%) during image acquisition. An Argon laser was used for ECFP (*l* = 458 nm) and EYFP (*l* = 514 nm) and a He-Ne laser for *l* = 633 nm. To determine the morphological parameters for the MTs network complexity, the total filament length, and the number of crossover points, the filament tracer option of the IMARIS Bitplane software has been employed. These two parameters are deduced by a software plugin that detects filamentous structures revealing information about the topology of filaments as the sum of the lengths of all lines and the number of crossover points within the filament. Images of aggregates have been acquired by Zeiss LSM 900 with Airyscan 2. For FRET experiments the sensitized emission approach has been employed. The donor ECFP was excited at 458 nm and its fluorescence emission was collected between 470 and 500 nm (donor channel) and between 530 and 600 nm (FRET channel). The acceptor EYFP was excited at 514 nm and its fluorescence emission was collected between 530 and 600 nm (acceptor channel). The donor and acceptor fluorophores were excited sequentially. The ImageJ software was used for image analysis. FRET images were corrected from cross-talk between the donor and acceptor channel using Youvan’s method: *F*_index = *I*_FRET — *A* × *I_D* — *B* × *I_A*, where *I*_FRET, *I_D*, and *I_A* are the images of the sample in the FRET, donor, and acceptor channel, respectively, and *A* and *B* are the fraction of the donor and acceptor leak-through into the FRET channel, respectively. The *A* and *B* bleed-through parameters were calculated by the ImageJ plugin FRET and Colocalization Analyzer. Values in our experimental conditions are *A* = 0.1 and *B* = 0.25, respectively. Normalized FRET (NFRET) was performed with the ImageJ software plugin “pixFRET” (Feige et al., 2005) by using:

NFRET = *F*_index/√ (*I_D* × *I_A*) (Xia and Liu, 2001).

FRAP experiments were performed by using the FRAP module coupled to the confocal microscope. For the pre-bleach phase, we acquired 10 frames of 512 × 512 pixel images at 1,000 Hz to define the initial level of fluorescence intensity. For the photobleaching phase, we selected a circular ROI with a radius of 2 μm in the cytoplasm of the cell. The photobleaching laser power was set at 50% for EYFP for five frames at 1,000 Hz. We set up the experimental parameters of acquisition to avoid whole-cell photobleaching. For the post-bleaching phase, 120 images have been recorded to follow the recovery of the fluorescence intensity in the selected ROI. Fluorescence recovery of the ROI was analyzed by the following steps: background subtraction; first normalization to the initial pre-bleach value of fluorescence intensity; correction for the fluorescence loss; additional normalization to set the first post-bleach point to zero. At least 15 separate FRAP experiments for each sample have been performed. FRAP recovery curves have been fitted by a two-phase exponential association function.

### Statistical Analysis

Western blot experiments were analyzed by the non-parametric Mann–Whitney test. All results are shown as mean ± SEM from four independent experiments. For imaging experiments, statistical significance was assessed by ANOVA test and Student’s *t*-test. All results are shown as mean ± SEM from at least 15 samples. Significance is indicated as *p < 0.05, **p < 0.01.

## Results

### Q336H Determines a Bent Paperclip Conformation of Tau Increasing Its Stability on Microtubules

It is known that protein conformation of intrinsically disordered proteins such as Tau can be altered by molecular interactions with cofactors and binding proteins. We have developed the CST Tau biosensor, a genetically encoded FRET sensor based on the full-length Tau fused at the N-terminus with ECFP and at the C-terminus with EYFP. This tool can detect the conformation of Tau under different cellular conditions, including binding to interactors in live cells (Di Primio et al., 2017; Siano et al., 2019a). By using CST, we have previously reported that Tau assumes a relaxed conformation when it is soluble into the cytoplasm, while, upon binding to tubulin, it displays a paperclip conformation with the N-and C-termini in proximity (Di Primio et al., 2017).

**Figure 1:**
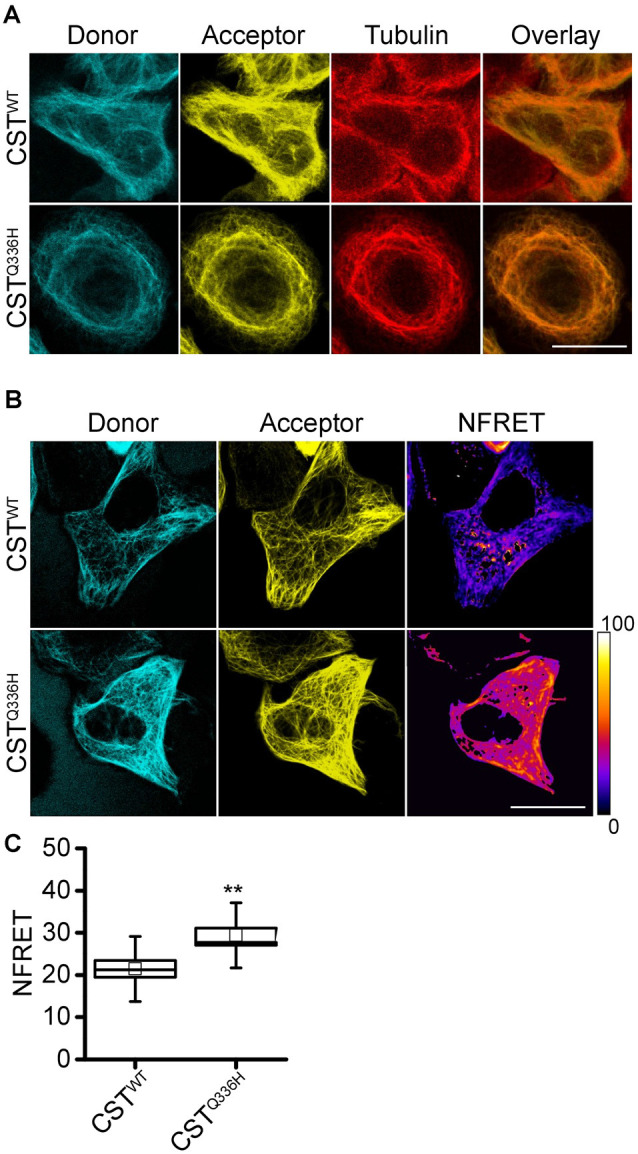
CST^Q336H^ conformation in live cells. **(A)** Imaging of HeLa cells expressing CST^WT^ or CST^Q336H^ and hybridized with anti-α-tubulin antibody; donor channel (blue), acceptor channel (yellow), and Tubulin (red). White scale bar = 10 μm. **(B)** NFRET images (false color), donor (Blue), acceptor (yellow) of reporter cells. White scale bar = 10 μm. **(C)** NFRET quantification of cells expressing CST^WT^ (*n* = 15), CST^Q336H^ (*n* = 21). Box spans the standard error of the mean, while whiskers indicate the standard deviation (*t*-Student’s test, **p < 0.01).

Here we employed the CST to investigate the impact of the pathological mutation Q336H. First, we inserted the CST^Q336H^ mutation in the CST^WT^ and we expressed it in Hela cells to study its binding to tubulin by immunofluorescence experiments. Figure 1A shows that the mutant Tau mainly localizes on MTs, very similar to the wild type.

To investigate whether Q336H mutation could impact 3D protein conformation we performed FRET experiments and we observed a significantly higher NFRET value compared to cells expressing CST^WT^ (CST^WT^: 21.44 ± 1.98; CST^Q336H^: 29.42 ± 1.68). This unexpected result suggests that this mutation alters Tau conformation reducing the distance between the N- and C-terminal ends of the protein (Figures 1B,C). Intriguingly, other pathological mutants (P301L and ΔK280) displayed a lower NFRET value compared to the wild type as previously reported (Di Primio et al., 2017).

### Q336H Increases Tau Stability on Microtubules

**Figure 2:**
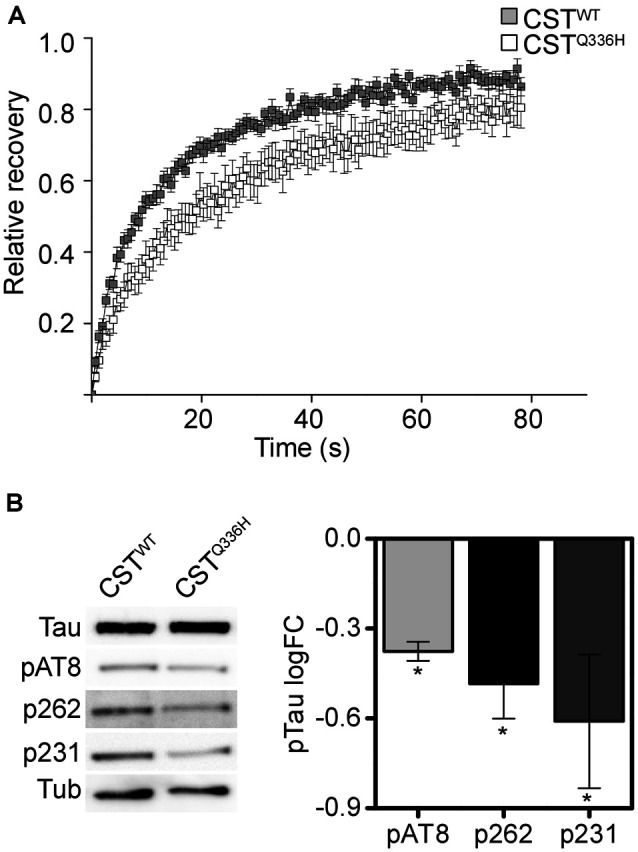
CST^Q336H^ mobility in live cells. **(A)** FRAP relative recovery curves of CST^WT^ and CST^Q336H^. **(B)** Phosphorylation profile of CST^WT^ and CST^Q336H^. Western blot analyses at specific pathological epitopes and relative quantification (Mann–Whitney test, *p < 0.05).

To investigate whether the Q336H mutation could alter Tau interaction with the cytoskeleton we checked its mobility by FRAP experiments. FRAP recovery curves showed that Q336H determines a reduction in the mobile fraction (Mob_calc_ CST^WT^: 89 ± 6%; CST^Q336H^: 79 ± 3%) with a substantial increase of the phase with lower mobility compared to CST^WT^ (named MTs-bound fraction CST^WT^: 61 ± 2%; CST^Q336H^: 81 ± 2%; Figure 2A).

The lower mobility indicates that Q336H mutation determines a stronger and more stable interaction with tubulin than the wild-type and this is presumably related to the particular conformation that it assumes on MTs.

The phosphorylation profile of Tau is a well-studied trait that indicates the affinity of Tau for microtubule binding, indeed, Tau hyperphosphorylation is thought to mainly contribute to detachment from MTs. We checked Tau phosphorylation levels at critical residues related to pathology: AT8, S262, and T231. The western blot analysis confirmed that Tau is partially phosphorylated under normal conditions, as expected. However, Tau^Q336H^ shows a paradoxically reduced phosphorylation level at the three epitopes (Figure 2B).

**Figure 3:**
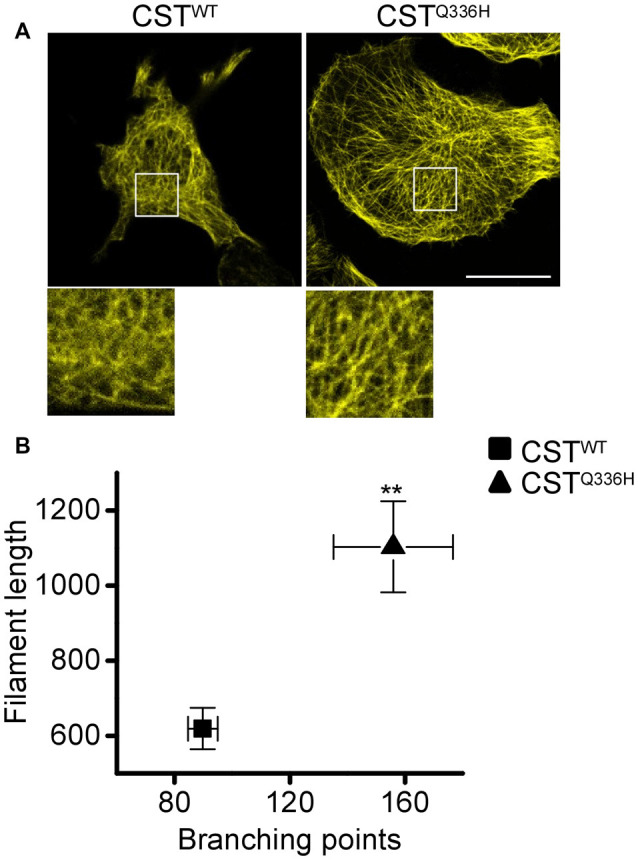
Impact of Q336H mutation on cytoskeleton complexity. **(A)** Confocal imaging of reporter cells (acceptor channel, yellow) expressing CST^WT^ and CST^Q336H^. White boxes are image magnifications. **(B)** Morphological analysis of fluorescent network density in reporter cells expressing CST^WT^ (*n* = 11), CST^Q336H^ (*n* = 13). Data shown are mean ± SEM (ANOVA test, **p < 0.01).

Previous work by Tacik et al. (2015) revealed that the Q336H mutation increases tubulin polymerization and stabilization *in vitro*. To understand if this mutation affects the cytoskeletal network complexity in live cells, we performed a network analysis on total filament length and branching points. The CST^Q336H^ expressing cells showed a significant increase in both parameters (Figure 3), indicating that this mutation is associated with higher complexity of the MT network. Remarkably, we previously reported that pathological mutations such as P310L and ΔK280 reduce the MTs network complexity compared to the wild-type. On the contrary, the AT8mut showed an increased MTs network complexity, higher stability, and a closed paperclip conformation similar to that of Q336H (Di Primio et al., 2017).

Overall, the stronger interaction with tubulin, the lower level of phosphorylation, and the increased complexity of the MTs network indicate that Q336H mutation has a stabilizing effect on Tau protein compared to other pathological mutations, studied so far, which generally favors Tau phosphorylation and detachment from microtubules.

### Q336H Increases Tau Propensity to Aggregation

**Figure 4:**
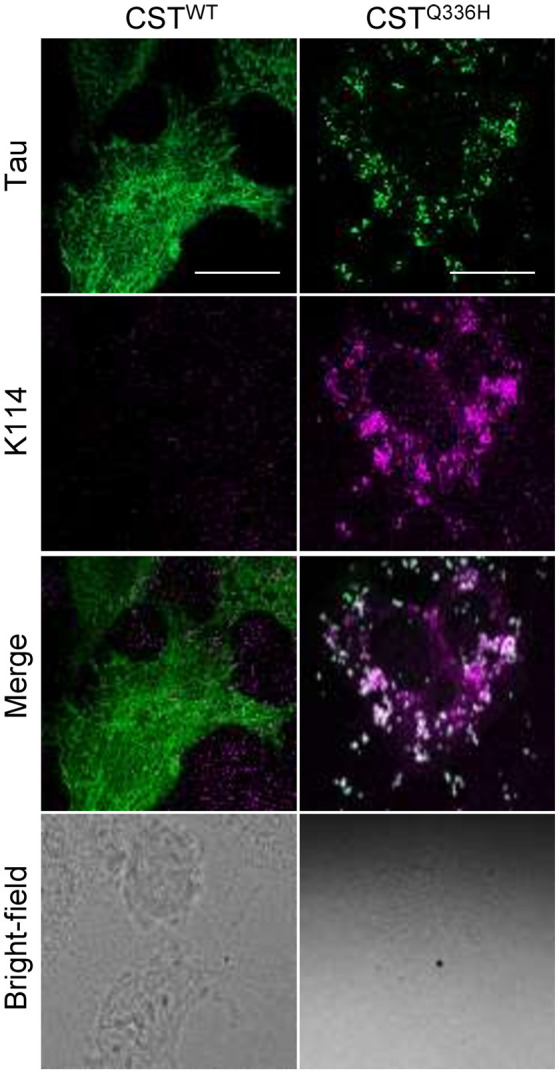
Impact of Q336H mutation on aggregation. Imaging of reporter cells 72 h after treatment with synthetic Tau seeds; Tau (green); K114 (magenta); bright field. White scale bar = 10 μm.

Despite Q336H mutation conferring particular properties that resemble a physiological rather than a pathological Tau protein, it causes hereditary PiD. To investigate the impact of Q336H on aggregation, we treated CST reporter cells with preformed fibrils for 3 days. For this assay, we employed P301S seeds that are a well-known and reliable reagent to prime Tau aggregation, and we demonstrated that cells expressing the CST^WT^ did not show any formation of Tau intracellular aggregates, as expected. On the contrary, cells expressing the CST^Q336H^ showed the presence of intracellular inclusions similar to aggregates from other pathological mutants (Siano et al., 2019a; Figure 4). To further verify whether CST^Q336H^ aggregates share the same amyloidogenic structure acquired by aggregates obtained from other pathological mutants, we stained cells with K114 to identify β-sheet amyloid structures.

Remarkably, CST^Q336H^ inclusions are positive to K114 demonstrating that this mutation is sensitive to aggregation as well as other mutants linked to tauopathies (Figure 4). This result suggests that even if in physiological conditions Q336H induces stabilization of Tau protein on microtubules, it also increases Tau sensitivity to aggregation, thus confirming its contribution to the PiD progression.

## Discussion

Here we report the characterization in live cells of a Tau pathological mutation involved in PiD (Tacik et al., 2015). Up to now, about 50 mutations have been identified to be associated with familial tauopathies and the majority occurs in the microtubule-binding domain (MTBD) that plays a central role in the regulation of microtubule polymerization. However, while some mutations alter Tau splicing and the 3R/4R isoform balance, the majority is involved in reducing Tau affinity and binding to MTs, thus causing cytoskeletal instability, Tau hyperphosphorylation, and aggregation (Goedert and Jakes, 2005; Wolfe, 2009; Strang et al., 2019). The current understanding point is that the pathological hyperphosphorylation causes a reduced interaction of Tau with microtubules and an increased concentration of soluble Tau, thus causing its aggregation (Alonso et al., 1997; Avila et al., 2004; Haase et al., 2004; Iqbal et al., 2010; Despres et al., 2017).

Two novel missense mutations in the MTBD, Q336R, and Q336H, have been identified in patients with familial PiD. The neuropathological and histological assessment revealed the typical features of Tau pathology including Tau hyperphosphorylation and NFTs (Pickering-Brown et al., 2004; Tacik et al., 2015). Remarkably, biochemical studies revealed the unusual behavior of Q336H/R mutations since they increase *in vitro* the MTs polymerization contrary to pathological mutations already described (Hasegawa et al., 1998; Hutton et al., 1998; Spillantini et al., 1998; Dayanandan et al., 1999; Mirra et al., 1999; Barghorn et al., 2000; Pickering-Brown et al., 2004; Goedert and Jakes, 2005; Fischer et al., 2007; Alonso et al., 2008; Tacik et al., 2015; Di Primio et al., 2017; Strang et al., 2019). However, no study reporting the properties of these Tau mutants in cells has been reported.

We introduced the Q336H mutation in our CST conformational Tau biosensor and expressed it in HeLa cells. We studied the conformation and the intracellular mobility of Q336H mutant and we reported that the paperclip fold that Tau usually assumes when it is bound to microtubules is even more locked in Tau^Q336H^ concerning the wild type. Moreover, this mutant is more stable on MTs as indicated by the reduced mobile fraction and the increased MTs-bound phase. In a previous study, we have already reported the tight correlation between Tau conformation and its affinity to MTs in live cells, indeed, soluble molecules display a relaxed conformation while Tau bound to tubulin fold with a paperclip conformation with the N and C-termini nearby (Di Primio et al., 2017). The more locked loop conformation caused by Q336H mutation significantly modifies Tau properties leading to higher stability on the cytoskeleton. Remarkably, Q336H displays opposite behavior concerning other pathological mutations such as P301L and ΔK280, which showed a relaxed protein conformation and higher mobility (Di Primio et al., 2017). Despite the resolution level of the CST system does not allow to establish a causal link between Tau conformation (FRET) and MT binding (FRAP), however, altogether these results indicate their tight association.

As a consequence, the Q336H mutant showed a particular impact also on the cytoskeletal organization. Indeed, while Tau molecules bearing well-studied mutations such as P301L or ΔK280 have low affinity to tubulin and cause MTs destabilization leading to lower complexity of the cytoskeletal network (Barghorn et al., 2004; Goedert and Jakes, 2005; Di Primio et al., 2017), cells expressing Q336H mutant show a network complexity higher than the wild type and the other mutants. This evidence demonstrates in living cells the previously reported ability of this mutant to increase tubulin polymerization, in contrast to the mutations discovered up to now. Moreover, it has to be noted that Tau also modulates microtubule dynamics (Bunker et al., 2004) and the increased microtubule stability could be harmful.

A reduction in the phosphorylation profile of the Q336H mutant might partially explain the increased stability on MTs. Indeed, hyperphosphorylation weakens Tau affinity to tubulin and is associated with Tau detachment from MTs and pathological aggregation (Pei et al., 2003; Iqbal et al., 2010). Interestingly, critical epitopes generally related to Tau pathology (AT8, p262, and p231; Sengupta et al., 1998; Braak et al., 2006; Alonso et al., 2010; Bibow et al., 2011) display reduced phosphorylation, suggesting that Tau^Q336H^ is less prone to phosphorylation. It is conceivable that the peculiar conformation of this mutant might alter/impede the interaction with kinases or might somehow hide the targeting epitopes.

Other molecular mechanisms might explain the increased stability on MTs such as local structural changes in the MTBD or changes in the local polarity. On the other hand, previous pieces of evidence on proximal mutations G335 and V337 underlie that this small domain of Tau (G335-Q336-V337) plays an important role in both microtubule binding and Tau aggregation and that mutations in this domain can lead to Tau pathology (Poorkaj et al., 1998; Spina et al., 2007, 2017). It would be interesting to investigate the contribution of these mutations in the context of CST.

Interestingly, even if its biophysical properties closely resemble more a physiological condition than a pathological one, the presence of Tau^Q336H^ in human brains leads to PiD with the formation of typical Tau aggregates (Tacik et al., 2015). In our assay, upon aggregation induction, cells expressing CST^Q336H^ showed the formation of intracellular inclusions suggesting that this mutation has a pro-aggregation effect as well as other pathological mutations. These aggregates resulted in K114 positive showing an amyloidogenic structure, however, since the ^336^QVEVK^340^ sequence targets Tau for degradation *via* chaperone-mediated autophagy (Wang et al., 2009) we cannot exclude the partial contribution of Tau accumulation at lysosomes for the aggregation induction.

The mechanisms behind the aggregation of this particular mutant are unclear and even more puzzling since strategies aimed at reducing Tau phosphorylation and increased stability on MTs are commonly considered as preventing traits for Tau pathology (Hanger et al., 2009; Bakota and Brandt, 2016). We hypothesize that this mutation might induce local changes in the MTBD conformation leading to a protein more prone to aggregate. The Q336 site and surrounding amino acids constitute a sensitive region of Tau for aggregation, indeed, it has been found in the structural core of aggregates in both Alzheimer’s disease and PiD by Fitzpatrick et al. (2017) and Falcon et al. (2018), respectively. It is quite conceivable that mutations at this site could promote aggregation of the protein (Fitzpatrick et al., 2017; Falcon et al., 2018). Alternatively, its peculiar conformation might alter the binding with cofactors or interacting proteins, thus enhancing Tau pathology. Another hypothesis is that the over-stabilization of microtubules could be toxic to neurons *per se*. Indeed, the MT dynamic is finely tuned and it is known that the uncontrolled stabilization of MTs induced by MTs stabilizing agents impair axonal trafficking and might compromise learning and memory (Chiorazzi et al., 2009; LaPointe et al., 2013; Atarod et al., 2015).

A caveat of this study is that the CST is a 4R-Tau construct that we used to model a 3R-Tau aggregation, therefore, this tool allowed only to monitor the aspects related to Tau conformation and destabilization.

However, altogether these results underline a dualism for the Q336H mutation that in physiological conditions is stable on MTs and enhances MTs polymerization but, once exposed to seeds, it supports and promotes toxic aggregation. Remarkably, the unique properties of this mutation also suggest the hypothesis that the mechanisms of Tau aggregation might be partially independent of its binding to tubulin or its phosphorylation state and that other unusual and still unexplored aspects such as structural alterations or cofactors might be responsible. Thus, our data demonstrate that Tau hyperphosphorylation and the ensuing reduced affinity for Tau can be dissociated from Tau aggregation in cells. This shows that the aggregation propensity of the Tau Q336H mutant in cells is not secondary to the increase of its concentration in the soluble pool, following hyperphosphorylation and detachment from microtubules, but has an intrinsic structural cause. Altogether, the data suggest that the Tau toxicity might be linked to the increased aggregation of Tau and not a loss of MT function. However, further experiments in more relevant cell lines (neuronal and glial) and in an appropriate *in vivo* model would clarify this point.

In any event, this result has important implications for the current therapeutic strategies aimed at reducing Tau hyperphosphorylation and promoting its interaction with microtubules and might point the attention to other non-canonical functions of Tau, such as, for instance, its recently described actions in the nucleus (Siano et al., 2019b).

The employment of Tau^Q336H^ in cell models and, above all, in transgenic mouse models, might help to identify pathological aggregation mechanisms still unexplored.

## Data Availability Statement

The original contributions presented in the study are included in the article, further inquiries can be directed to the corresponding author/s.

## Author Contributions

GS, CD, and AC designed experimentation and wrote the manuscript. GS, MM, AS, and VQ performed the experiments. GS, MM, and VQ collected and analyzed the data. MM, AS, and VQ contributed with the discussion and correction of the manuscript. All authors contributed to the article and approved the submitted version.

## Conflict of Interest

The authors declare that the research was conducted in the absence of any commercial or financial relationships that could be construed as a potential conflict of interest.
